# Editorial: Pediatric Central Nervous System Tumors: State-of-the-Art and Debated Aspects

**DOI:** 10.3389/fped.2020.00091

**Published:** 2020-03-10

**Authors:** Andrea Carai, Angela Mastronuzzi

**Affiliations:** ^1^Neurosurgery Unit, Department of Neuroscience and Neurorehabilitation, Bambino Gesù Children's Hospital, IRCCS, Rome, Italy; ^2^Neuro-Oncology Unit, Department of Onco-Hematology, Cell and Gene Therapy, Bambino Gesù Children's Hospital, IRCCS, Rome, Italy

**Keywords:** brain tumors, pediatric neuro-oncology, children, central nervous system tumors, pediatric oncology

Central nervous system tumors are the second most frequent malignancy in children and young adults. Despite this, they remain rare conditions and management standardization continues to be challenging despite international networking efforts.

The Research Topic on “pediatric central nervous system tumors: state-of-the-art and debated aspects” we included innovative and original contributions on multiple aspects of pediatric neuro-oncology.

In reference to the neuroimaging, the work of Colafati et al. presents preliminary data suggesting direct involvement of the V cranial nerve at diagnosis as a negative prognostic marker in DIPG.

In their analysis, 13.8% of the children presented cranial nerve V involvement at diagnosis. This finding was associated with a poor prognosis (median overall survival: 7 vs. 13 months), concluding that cranial nerve V should be routinely evaluated with diagnostic scans.

Even if these findings need to be confirmed with a larger series, it is important to notice that, in the era of radiomics, accurate interpretation of traditional MR sequences can still contribute in advancing clinical knowledge.

In the paper by Yalon et al. an elevated neutrophil to lymphocyte ratio, which is a relatively simple blood-derived biomarker, is suggested to be a hallmark of malignant brain tumors. The biological justification for this observation would be both a reduction in lymphocytes, considered to be protective from cancer, and an increase in neutrophils, usually associated to tumor progression. This paper highlights two promising fields of pediatric neuro-oncology: the potential role of immunity modulation for treatment and the opportunity offered by the development of biomarkers to assist in the treatment of patients.

The contribution by Foster et al. offers a wide overview on the advancement of neuro-oncology surgery. Sophisticated techniques allow more accurate surgical planning, better visualization and orientation during surgery, and an increase of intraoperative safety. Advancing the possibilities in tumor resection while preserving neurological functions, will certainly overall contribute to better treatment results, specifically for patients with low-grade lesions that still suffer significant surgical morbidity.

In the last decades, significant progress in oncology has been achieved through molecular characterization of tumors and targeted therapies. Unfortunately, not all tumor types are eligible for these treatment options. into It is critical for the treatment to detect if the BRAF V600E mutation is found in a subset of pediatric low-grade gliomas, thus specific inhibitors of the mutated protein, such as Vemurafenib, can be used. In the paper by Del Bufalo et al., the authors investigated Vemurafenib's safety and efficacy as a single agent in pediatric patients with BRAFv600E positive LGG, which showed very encouraging results.

Petruzzellis et al. further demonstrated the efficacy and safety of this target therapy, in the setting of Pleomorphic Xanthoastrocytoma (PXA) associated with Down syndrome. PXA is a rare WHO grade II tumor that can harbor the BRAF mutation p.V600E. This case report describes the first occurrence of a PXA reported in a child with Down syndrome (DS) as well as the first use of Vemurafenib in DS. The treatment was well-tolerated, and the efficacy was seen by a partial response and a stabilization of the disease. In conclusion, despite the use of Vemurafenib, not yet standardized for pediatric patients affected by brain tumors and DS, we have shown the feasibility of this therapeutic approach.

In addition to the molecular characterization of tumors, several significant discoveries have contributed to shedding light on the role of epigenetic modification and cellular microenvironment in tumor growth and progression. Proteins of the Polycomb group (PcG), which is one of the major epigenetic modification, can be differentiated in polycomb repressive complexes (PRCs): PRC1 and PRC2. The trimethylation of lysine on Histone H3 is an epigenetic modification induced by enhancer of zeste homolog 2 (EZH2), the catalytic core subunit of PRC2, leading to the silencing of many tumor suppressor genes. Overexpression of EZH2, evidenced by a growing number in data, is associated with a poor outcome and progression in a large number of cancer cases.

Hypoxia inducible factor (HIF), a crucial transcription factor involved in promoting and regulating tumor development, promotes inflammation, angiogenesis, metabolic reprogramming, invasion, and metastatic fate.

In their review, Papale et al. analyzed the activity and influence of EZH2 and HIF in pediatric cancer progression, the correlation between them and the possible future role of specific inhibitors.

Medulloblastoma is among the most common malignant childhood brain tumors (WHO grade IV). Genomic studies have defined four consensus molecular subgroups (WNT, SHH, Group 3, and Group 4), each are characterized by distinct clinical outcomes, copy-number variation, transcriptional profiles, and somatic mutations.

Aberrant expression of long non-coding RNAs, which are normally expressed in the human brain, have been linked to neuro-oncological disorders. In their paper, Laneve et al. tried to explain the function of long non-coding RNAs in the medulloblastoma biology and development.

From another point of view, several studies have investigated the role of epigenetic modulators in various types of cancers. Recently, the molecular epigenetic deregulation in Medulloblastoma has been reviewed, highlighting the pathways implicated in the disease, their different biological behaviors and possible future target therapies. In this setting, Zwergel et al. have summarized and highlighted epigenetic modulators as promising drug targets in MB.

Moreover, Abballe et al. investigated the role of Numb in medulloblastoma's cancer cells. Their study showed that Numb p66, which is expressed in medulloblastoma stem-like cells and cerebellar neuronal stem cells (NSCs), modulates cancer staminality. In particular, the medulloblastoma samples analyzed in this study, showed low levels of Numb p66 and overexpression of Numb p72 compared to normal tissue. These results show different roles for the two major Numb isoforms evaluated in medulloblastoma, which highlighted a central role for Numb p66 in regulating stem-like cells and NCS maintenance.

Pediatric neuro-oncology remains a challenging arena for researchers with different expertise. We believe that the coordinated work on the study of different types of tumor, as found in this Research Topic, from various points of view, will be vital in the contribution to advancing the knowledge of these tumors, will be key to the improvement clinical results.

## Author Contributions

AC and AM have jointly contributed intellectually and materially to the work, and approved for publication.

### Conflict of Interest

The authors declare that the research was conducted in the absence of any commercial or financial relationships that could be construed as a potential conflict of interest.

